# Occupational Exposure to Blood and Body Fluids in a Department of Oral Sciences: Results of a Thirteen-Year Surveillance Study

**DOI:** 10.1155/2013/459281

**Published:** 2013-02-14

**Authors:** M. R. A. Gatto, L. Bandini, M. Montevecchi, L. Checchi

**Affiliations:** ^1^School of Dental Hygiene, “Alma Mater Studiorum” University of Bologna, 40125 Bologna, Italy; ^2^DIBINEM, “Alma Mater Studiorum” University of Bologna, 40125 Bologna, Italy

## Abstract

*Objectives.* Aim of this analysis was to identify trends that will aid in the prevention of injury. *Methods.* Our data were collected from 1999 to 2011 during a surveillance program of occupational exposures to blood or other potentially infectious materials in a Dental School by using a standard coded protocol. *Results.* 63 exposures were reported. 56/63 (89%) percutaneous and 7/63 (11%) mucosal, involving a splash to the eye of the dental care workers (DCW). 25/63 (40%) involved students, 23/63 (36%) DCW attending masters and doctorate, 13/63 (21%) DCW attending as tutors and 2/63 (3%) staff. 45/63 (71%) and 18/63 (29%) occurred respectively during and after the use of the device; of last ones, 1/18 (0.05%) were related to instrument clean-up and 1/18 (0.05%) to laboratory activity, 12/18 (67%) occurred when a DCW collided with a sharp object during the setting, and 4/18 (22%) during other activities. The instrument and the body part most likely involved were needle and finger respectively. The overall exposure rate was 4.78 per 10,000 patient visits. Conclusions Our results may serve as benchmark that Dental Schools can employ to assess their frequency of injury.

## 1. Introduction

On job safeness the Italian law sets common principles for private and public health care structures [1–3]. The health manager is recognized as the responsible for the employees' safety and is called to provide a safe work environment ensuring full protection from job hazards [4–6].

In spite of a rigorous respect of these indications, the accident can anyway occur influenced by the human factor or unpredictable events.

At a dental school a considerable share of teaching time is dedicated to clinical activity, with high probability of direct and indirect accidents, as the student can be considered more prone to possible accident than experienced operator.

The dental school has a primary role on current and future safeness of the student. It must offer the best protection and survey but also, it must form the student's risk perception and safe behaviour, which can be induced only through practical experience. During this teaching time the faculty is called to a rigorous survey and respect of the operative protocols in order to prevent eventual ramifications.

Many people are involved in the university clinical activity: faculty, staff members, tutors, students and professionists attending postgraduate courses.

This surveillance study aims to report clinical and nonclinical injuries that occurred in the Department of Oral Sciences (DS) of the “Alma Mater Studiorum” University of Bologna over a thirteen-year period (1999–2011), to identify trends and evaluate their relevance to the procedures performed during the clinical activity, with the final scope of evaluating their risk of occurrence and determine if additional safety precautions are needed or if modification of current procedures might be indicated. 

## 2. Material and Methods 

The incidence and the characteristics of the injuries occurred over a thirteen-year period (1999–2011) at the dental school of the University of Bologna were collected and analyzed.

Following a previous work [7], subjects involved were classified into faculty that includes professors and researchers, staff that includes dental assistants, nurses, and executive assistants, students, and other personnel represented by dentists attending postgraduate courses.

The mean number of people evaluated was of 335 subjects per year, divided into 45 members of the staff (age range: 25–70 y), 190 students (age range: 18–23 y), and 100 other personnels (24–53 y).

Depending on the occurrence time, the accidents were divided into clinical and nonclinical; a clinical one was considered each of injury occurred during the patient treatment.

No accidental injury caused by fall or collision occurred in the period examined.

In order to compare the number of injuries with clinical activity, the number of dental treatments per year was collected. 

For each accident were recorded the following variables:

- sex and age of the operator involved,
- typology of the injured subject (faculty, staff, student, and other personnel),
- category of accident (clinical or nonclinical),
- body part injured: eyes, head, right or left hand (thumb, other fingers, and palm),
- date and time of accident,
- physical location of the incident: treatment areas, teaching areas,
- instrument causing the injury: anaesthetic needle (carpoule, disposable carpoule, and peripress), solid needle, (suture), irrigation needle or periodontal instrument (explorer, scaler, curette, knife, and probes), bur, scalpel blade, scissor, endodontic instrument (file, explorer) ultrasounds, blades, contaminated vial, splash.
- serological examination to ascertain infection by HVB, HVC, HIV of the patient involved.

### 2.1. Statistical Analysis

Univariate techniques were used to describe the data: frequency distribution and pie-chart for nominal variables, median and range for quantitative variables, and column chart for the prevalence of accident per year. *χ* ^2^ test was used to evidence the associations between nominal variables. Bivariate linear regression was used to demonstrate the presence of a trend between the number of patient visits and the number of accidents; the association between the two variables was verified by means of Spearman's rho coefficient. *α* level was a priori set at 0.05. 

## 3. Results

 There were 63 incidents within DS from 1999 to 2011 and 131699 patient visits (the minimum patient visits in 1999 was 8661 and the maximum in 2007 was 117199 with a mean of 10131 treatments per year); the rate between the number of incidents to the number of visits was of 1 injury to 2090 visits; the incidence/10000 visits ± standard error was 4.78 ± 0.60 all over the period considered.

 The number of operators involved was 61, 28 males (45%) and 33 females (55%); no significant difference was observed between sexes. Double injuries occurred to 2 operators were of different types and happened in different times.

The age of the damaged operators was between 19 and 60 years (median age: 26 years). 

**Figure 1:**
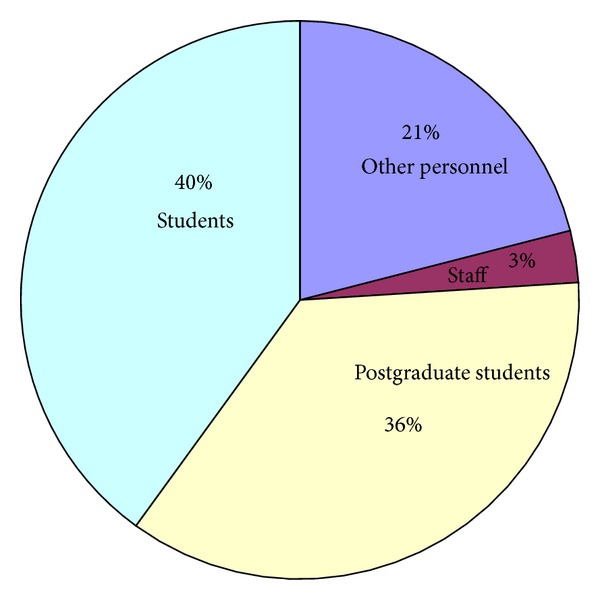
Percentage of accidents by operator category.

40% of those involved in the accidents were students and 36% dentists attending M.S. and PhD studies (Figure 1).

**Figure 2:**
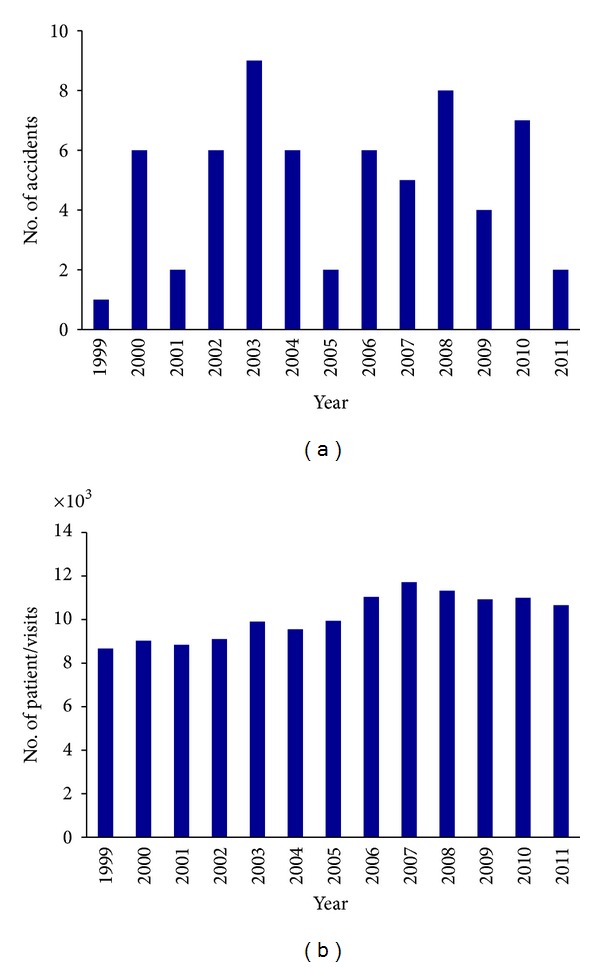


Considering the number of injuries per year (Figure 2(a)) it emerges that it reaches a peak in 2003 (9 injuries) and the minimum level in 1999 (1 injury). It is interesting to observe that the number of clinical activities increases proportionally from 1999 to 2011 (*p* = 0.0001) (Figure 2(b)) while the number of injuries does not follow this trend (*p* = 0.6409). No statistically significant association is observed between the number of clinical activities and the number of injuries (*p* = 0.217).

The highest number of injuries happened in June and November (*n* = 8) and the minimum in July and December (*n* = 3). The month of August was not considered because the clinical activity is regularly suspended.

The highest number of injuries happened at the week beginning (16 on Monday and 15 on Tuesday) while the minimum on Wednesday (8 of 63, 13%). No clinical activity is carried out on Saturday and Sunday.

Almost all the injuries happened from 9.00 AM to 13.00 AM (95%), divided into equal numbers from 9.00 AM to 11.00 AM (*n* = 29) and from 11.00  AM to 13.00  AM (*n* = 31).

The highest number of injuries was clinical (45 of 63, 71 %), 18 (29%) were not clinical and took place prevalently during instrument setting (12 of 18, 67%), only one was in laboratory and during cleaning procedures (0.05%) and 4 during other activities (22%).

As for the relationship between activity and type of operator, the 33% of the clinical injuries involves students and such percentage goes up to 50% for the clinical injuries (chi-square = 66.91, *p* = 0.0001). 

**Figure 3:**
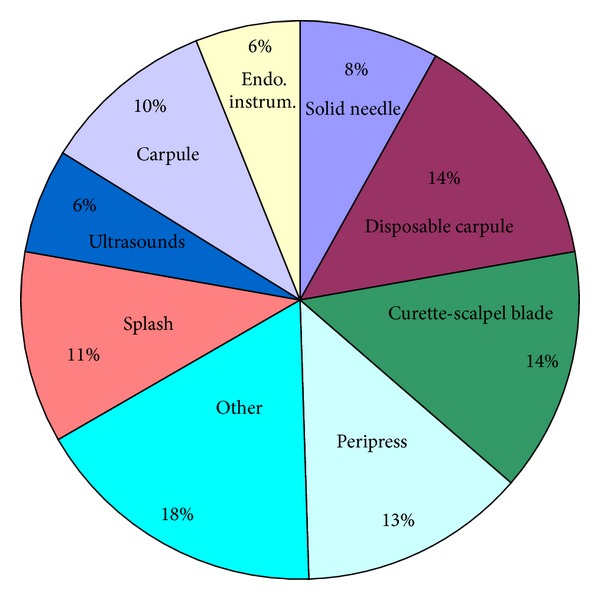
Instruments causing accidents, as percentages.

The instrument more frequently connected to the accident was the needle for local anaesthesia (23 of 63, 36.5%) (Figure 3).

No statistically significant association was observed between the instrument involved and professional profile of operator or type of activity.

The injuries involved the hands for 78%, 11% the eyes and 2% the head (Table 1); no significant differences appears among right- and left-hand sites.

A significant association is observed between the cause of injury and the part of body involved (*p* = 0.0001); right thumb is injured by carpule (23%), the other fingers of the right hand by carpule (31%) and curette or scalpel blade (50%), the eyes by blood or noun (100%), and the left thumb by peripress needle (29%). 

 Since 2002, date of its institution, endodontic division presented the highest number of injuries (15 of 62, 24%), respectively followed by the teaching section (12 of 62, 19%), periodontology and implantology (11 of 62, 18%), oral surgery (10 of 62, 16%), and microbiology and other services (both 1 of 62, 2%). No significant association was found between section and number of injuries or instruments involved.

 The 22% of patients involved in the injury event, regularly submitted to a serologic examination, resulted positive to HVB or HVC or HIV (1 HBV positive, 10 HCV positives, and 3 HIV positives); however no seroconversion happened within 6 months from the injury neither in students nor in staff.

## 4. Discussion

Within the DS all the laws that rule the health departments for the prevention of occupational injuries were activated [2]. Particularly guidelines aimed to avoid needle or sharp instrument or contamination injury occurring during patient treatment or instrument reordering were adopted. [8, 9].

Faculty and staff members are instructed to follow the security guidelines in workplace; also the students follow specific lessons before the beginning of the clinical activity.

When the incident happens for casualty notwithstanding the guidelines were followed or for insufficient application by the operator, each incident was reported to the staff member supervising clinical operations; each information was recorded and the appropriate action was initiated. In an emergency situation, the proper measures were taken to limit further injury [10, 11]. 

This paper reports all the injuries occurred from 1999 to 2011, completing data collection until 2008, previously published [12]. Although also in the previous years DS adopted procedures aimed to control crossinfections, particularly caused by needlestick [9], only from 1999 the procedures were standardized and a responsible for the safeness and an archive from which our data derived were created [10]. 

During these 13 years DS was interested in substantial and structural changes of management. The passage from a clinical to a departmental setting [13] has modified the management procedures that influenced also the planning of the clinical activity. The opening of new educational careers (M.S. studies, high specialty courses, PhD) enlarged the areas of interest of DS to fellow dentists coming from outside; the participation to Socrates-Erasmus programme has contributed to give an international relevance to DS by means of contacts with universities with different cultural backgrounds. The transformation of the School for Dental Hygienist from 2 year diploma on first level degree (3 years) brought to an increase of the number of students exposed to occupational risk [14].

The total number of incidents in the considered period was of 63, with a rate to the number of visits equal to 1 incident to 2090 visits. The number of visits was derived from the number of invoices, which are surely less than the number of visits because after some clinical procedures (suture removal, adaptation of a prosthesis, and controls) during an ongoing treatment not always an envoice is emitted. 

Numerous papers have reported the incidence of injuries in dental schools [15–19]; despite the different methods used to describe the data (incidents/year [20], rate/100 person/year [19], incidents/10000 patients visits [19], incidents/1000 activities [20], and mean number of incidents/20 days [21]) make it difficult to compare them.  Table 2 reports incidence/10000 patient visits found in studies carried out in dentistry practices. 

Our data do not permit to estimate the incidence rate per 100-year-person, being that the number of operators greatly varied during the period of observation. 

The highest number of incidents occurred in 2003 may be explained by the opening in that year of M.S. studies and high specialty courses with the consequent increase of the number of operators. 

The hypothesis that from 1999, with the adoption of specific procedure of prevention, the attention of the operators to the injuries following the exposition to biologic risk is been increasing, does not seem to be confirmed by data collected in 2008 when the number of incidents reached a peak in comparison to the previous years.

 June when the highest number of events happens is the last month of work before the summer holidays; therefore a greater physical and psychological fatigue of the operators with a consequent decrease of the attention might explain this result. 50% of the injuries occurred in the first two days of the week; also the interruption of the work during the week-end might provoke a decrease in the “emotional tension” in the performance of the work. The distribution of the injuries during the 24 hours respects the timetable of the clinical activity that is carried out mainly in the morning and only for a little part in the afternoon. 

The greatest number of accidents was of a clinical type (71%), while 29% was of nonclinical type.

One injury involved a staff member, confirming the importance of a greater experience. Staff is more motivated towards the job safeness because they participated in the definition of the procedures and guidelines used. A scarce work experience might explain the greater frequency of injuries both clinical and nonclinical, observed in the students and in the remaining personnel. The higher number of occurrences among the students is during the disposal of the instruments, that they make when they perform treatments while this operation that, when the treatment is carried out by the other odontoiatric personnel, the nurses are responsible for. 

The instruments more often involved are needles (36.5%); from the comparison between not disposable and disposable needles it emerges that the introduction of the last ones in the clinical practices has not reduced the number of occurrences. Other authors [7, 19, 22–28] report percentages between 31% and 45% for the injury from needles. 

Incidents caused by curettes and scalpel blades observed in our research are 15%. Other authors [15, 25, 28–31] report that scalpel blades are responsible for injuries from 8% to 26%. In other studies injuries caused by scalers and curettes are observed in percentages between 8% [25] and 12% [19]; injuries caused by root canal instruments and suture needles (6% and 8%, resp., in our study) are more frequent than in other researches (resp., 5% and 1%) [19].

The part of body more often involved is represented by the hands; our results agree with another paper [7] where both clinical and nonclinical occurrences involve the fingers and above all the thumb (resp., 25% and 45%).

It is notable that an adequate prevention could have avoided only eye injuries by means of the adoption of glasses and protective screens. 

The endodontic section, reported a percentage of accidents equal to 24% since 2002 when the section was instituted; however in this section clinical activities are performed also in the afternoon with the consequence of a greater number of hours of potential of risk. 

The prevention activity carried out in the DS concerning the infection from HIV and HBV has brought on the definition of guidelines [32] followed from the personnel since their production. Positivity towards HIV and other viruses with liver tropism seems to be an indicator of the prevalence of seropositivity in the patients of DS. From some studies [33–35] it emerges that the association between dentistry treatments and transmission of HIV is relatively small (from 0 to 0.08%). Seropositivity to HIV in our study is 6%; other authors report on values between 12% [19] and 17% [25]. Seropositivity to HCV (16%) observed in our study underlines the role of this virus, considered the most important amongst those with liver tropism because of its ability in inducing chronic infection in 85% of the infected [36–39]. 

Seropositivity to HBV in our study is 2% lower than 9% reported in a similar research [40].

During our period of surveillance no seroconversion has been verified as evidenced also by other authors both in dentistry [19, 25] and in other medical branches [41]; data from an Italian study [42] do not report on any conversion for HBV, percentages between 0.36% and 0.39% for HCV, and between 0.14% and 0.43% for HIV.

## 5. Conclusions

- Guidelines of occupational safeness adopted in the DS of the “Alma Mater Studiorum” University of Bologna seem to be effective given that the incidence/10000 patients visit is 4.78. Injuries concern mainly students in dentistry denoting the necessity, during the studies, of upgrading the level of knowledge on prevention of accidents, which would, at least partially, influence their reduction. Moreover, the instructors should monitor if the students are taking the necessary preventive measures without fail: the high number of injuries provoked by needles needs a greater respect of the procedures of their disposal.
- Seropositivity to HBV, HCV, and HIV was equal to 22% of patients involved in accidents however no seroconversion was registered in the operators who came in contact with these patients. These important data are derived from a procedure that predicts the adoption of control measures automatically: notification, control of the seropositivity of the patient and of the operator potentially infected. In the last thirteen years the DS has activated collaborations with a university teaching hospital (Policlinico Universitario S.Orsola-Malpighi) and at-risk patients (haemofilic, transplanted, and immunodepressed) have been treated. Consequently the adoption of standardized prevention procedures and the control of their application in clinical dentistry setting aimed to avoid incidents and, when they happened, to reduce the consequence for the operators, should be an important operative objective of a DS.

## Figures and Tables

**Table 1:** Parts of body affected by accident.

Part of body	*N*	%
Left hand	26	41
Thumb	6	23
Other fingers	15	58
Palm	5	19
Right hand	23	37
Thumb	6	26
Other fingers	15	65
Palm	2	9
Eyes	7	11
Head	1	2
Other part	6	9

**Table 2:** Incidence of accidents in the school of dentistry.

Authors	Study design	Incidence/10000 visits
Cleveland et al., 1995 [15]	Observational study (6 months)	12.5
Ramos-Gomez et al., 1997 [25]	Prospective study (5 years)	3.53
Kennedy and Hasler, 1999 [21]	Observational study (1 year)	4 (3rd-4th year students) 1.30 (staff)
Younai et al., 2001 [19]	Surveillance study (10 years)	3.59 (during 1994–1997)
Callan et al., 2006 [7]	Report (2 years)	5.24
Our paper [12]	Surveillance study (10 years)	5.15
